# Delayed Retrieval of a Patent Foramen Ovale Occluder From the Abdominal Aorta: Management and Challenges

**DOI:** 10.1016/j.jaccas.2025.106237

**Published:** 2025-12-11

**Authors:** Ruxandra I. Sava, Fabien Doguet, Isabelle Danjon, Igor Platonov, Dominique Fourchy, Davy Huel, Noémie Tence, Eric Durand, Hélène Eltchaninoff, Philippe Garot

**Affiliations:** a Institut Cardiovasculaire Paris-Sud, Hôpital Privé Jacques Cartier, Massy, France; b Abbott Cardiovasculaire, Rungis, France; c Service de Cardiologie, Centre Hospitalier Universitaire Charles Nicolle, Rouen, France

**Keywords:** endovascular retrieval, PFO prosthesis embolization

## Abstract

**Background:**

Patent foramen ovale (PFO) closure is an established strategy to prevent recurrent stroke from paradoxical embolism. Occluder embolization is a rare but serious complication.

**Case Summary:**

A 55-year-old man presented with abdominal discomfort 6 months post PFO closure. Transthoracic echocardiography failed to visualize the occluder, and a computed tomography scan revealed device embolization into the abdominal aorta. The patient underwent percutaneous retrieval of the device coupled with repeat PFO closure. Although the procedure was technically successful, he developed type B aortic dissection, which was successfully managed conservatively.

**Discussion:**

Delayed retrieval of a PFO occluder embolized into the aorta is associated with significant risk of aortic injury, regardless of endovascular or surgical approach.

**Take-Home Message:**

Adequate PFO sizing is critical to prevent device embolization. When endovascular retrieval is necessary, the use of a long sheath 2- to 5-F sizes larger than the original introducer is recommended.

## Graphical Abstract

**Figure:**
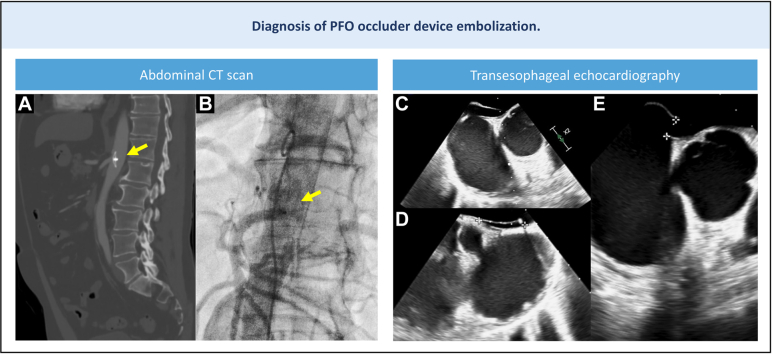

## History of Presentation

**Figure 1:**
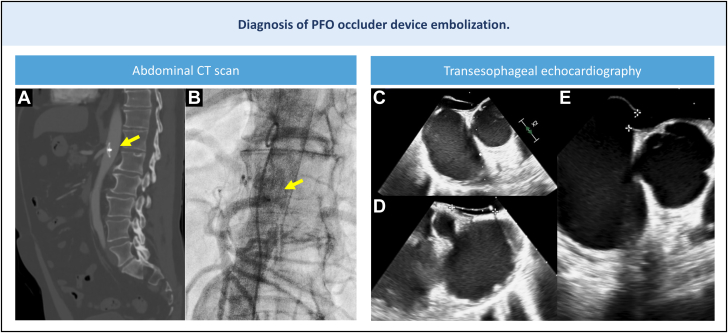
Non-invasive Imaging Demonstrating PFO Occluder Embolization (A and B) PFO device embolized into the abdominal aorta, depicted on CT scan and angiography (arrows). (C to E) TEE showing a large PFO. CT = computed tomography; PFO = patent foramen ovale; TEE = transesophageal echocardiography.

A 55-year-old man was referred to our center for percutaneous retrieval of a patent foramen ovale (PFO) occluder that embolized into the abdominal aorta. An Occlutech device with a 23-mm left atrial disk and a 25-mm right atrial disk had been implanted without complication. The device was confirmed in place on transthoracic echocardiography 24 hours post procedure; however, at the sixth month of follow-up, the device was no longer visualized, and a significant interatrial shunt was noted. The patient reported vague abdominal discomfort. A thoracoabdominal-pelvic computed tomography (CT) identified the occluder lodged at the level of the celiac trunk, between the T12 and L1 vertebrae (Figures 1A and 1B). Given the patient's symptoms and thrombotic risk, and considering the challenges of surgery, the heart team elected to proceed with percutaneous retrieval.

## Past Medical History

The patient had a body mass index of 26 kg/m^2^. He had experienced a lateral medullary stroke in July 2023. Apart from a significant PFO with right-to-left shunt, the diagnostic workup was unremarkable. A family history of aortic dissection in the patient's brother was noted.

## Differential Diagnosis

The patient's bowel function was normal, and physical examination was unremarkable. Abdominal CT showed no alternative cause for the abdominal discomfort aside from the occluder, which was located near the mesenteric artery and may have been causing transient mesenteric ischemia.

## Investigations

The electrocardiogram on admission showed sinus rhythm, without conduction or repolarization abnormalities.

The transthoracic echocardiography demonstrated a hypermobile interatrial septum with severe right-to-left shunting. Left ventricular function and dimensions were normal, as well as valvular function. The aortic valve was tricuspid, and the ascending aorta was mildly dilated at 42 mm.

The transesophageal echocardiogram (Figures 1C to 1E) showed a large PFO (Video 1), with a PFO tunnel length of 20 mm, width of 15 mm, and septal excursion of 16 mm. A bubble study revealed a grade 4 atrial shunt.

## Management (Medical/Interventions)

**Figure 2:**
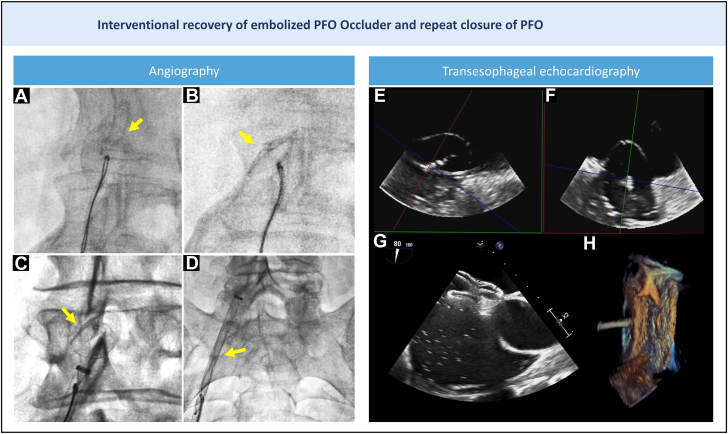
Angiography and TEE Guidance for Embolized Occlude Recovery and Repeat PFO Closure (A to D) Device (arrows) mobilization and retraction into a 14-F sheath by snaring with a tulip snare inside a Judkins right 6-F catheter. (E and F) A stretched diameter of 22 mm was measured by balloon sizing. (G) Complete abolition of right-left shunt as demonstrated by bubble testing. (H) Three-dimensional TEE showing adequate device positioning. PFO = patent foramen ovale; TEE = transesophageal echocardiography.

A right femoral arterial approach was used. A 28-mm long, 14-F Sentrant sheath (Medtronic) was positioned in the proximal right iliac artery. Initial attempts to mobilize the device using a pigtail catheter were unsuccessful. A 25-mm Amplatz Goose Neck snare (Medtronic) also failed to secure the device. A Tulip snare (En Snare, Merit Medical) introduced via a 6-F Judkins right catheter finally succeeded in anchoring the occluder. With steady traction applied to the snare-catheter unit, the prosthesis was mobilized (Figures 2A and 2B, Video 2). Hemodynamics remained stable under conscious sedation, and angiography showed no evidence of aortic injury (Video 3). The occluder was safely withdrawn into the sheath and fully externalized (Figures 2C and 2D, Videos 4 and 5).

**Figure 3:**
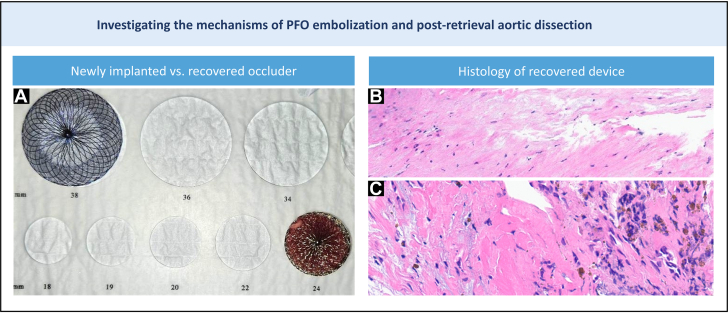
Mechanistic Insights into PFO Occluder Embolization and Aortic Dissection Following Endovascular Retrieval (A) We chose a 24-mm septal occluder ASD-type device (top left corner) for repeat PFO closure, which is much larger than the prior implanted 23 × 25 mm Occlutech PFO Occluder (bottom right corner). (B) H-E stain showing intimal growth on prosthesis. (C) Foreign-body granuloma. ASD = atrial septal defect; H-E = hematoxylin and eosin; PFO = patent foramen ovale.

A repeat PFO closure was then performed. Transesophageal echocardiogram showed a large PFO (Figures 1C to 1E). Balloon sizing (Figures 2E and 2F) revealed a stretched diameter of 22 mm, prompting the selection of a 24-mm Septal Occluder (Abbott), a device typically used for atrial septal defect closure. This was significantly larger than the initial Occlutech Occluder (Figure 3A). The new device showed excellent apposition, and postdeployment bubble testing was negative (Figures 2G and 2H, Video 6).

## Outcome and Follow-Up

**Figure 4:**
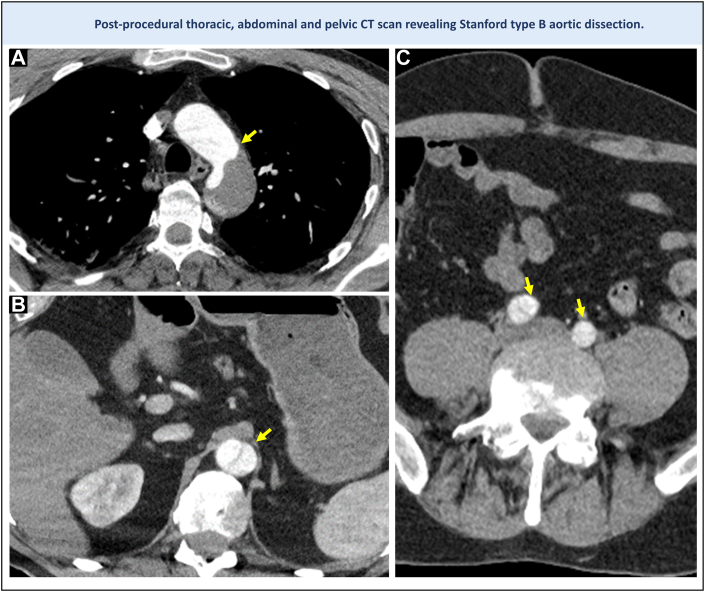
Computed Tomography Revealing a Serious Complication of Endovascular Occluder Recovery (A) Abdominal computed tomography demonstrating a dissection flap (arrow) at the level of the aortic arch, distal to the subclavian artery. (B) Aortic dissection originating at the site of occluder embolization (arrow). (C) Aortic dissection extending distally to both iliac arteries (arrows). PFO = patent foramen ovale.

Four hours after the procedure, the patient developed lower abdominal pain radiating to the chest. CT imaging revealed a type B aortic dissection (Figure 4), extending proximally from the abdominal aorta to the distal thoracic aorta, and distally to the common iliac arteries. In the absence of signs of ischemia, he was managed conservatively. A repeat scan at 1 week showed no progression, and he was discharged on day 10.

Histological examination of the explanted occluder revealed endothelialized fibrous tissue resembling neointima and a granulomatous foreign body reaction (Figures 3B and 3C), but no myocardial elements. These findings suggest the occluder had adhered firmly to the aortic wall, likely having embolized shortly after the initial procedure.

One year later, the patient remained clinically stable. He experienced an episode of symptomatic paroxysmal atrial fibrillation within the first month, successfully managed by bisoprolol (5 mg daily) and a non–vitamin K oral anticoagulant. No arrhythmia was detected on 24-hour electrocardiogram monitoring at 12 months.

## Discussion

This case demonstrates that delayed retrieval of an embolized PFO device from the abdominal aorta is feasible but carries substantial risk of aortic injury.

Device embolization is uncommon, with a reported incidence between 0.6% and 1.2%.^1^ Recognized risk factors include long PFO tunnel length and atrial septal aneurism.^1^^,^^2^ A literature search on PubMed using the query [(patent foramen ovale) AND ((prosthesis) OR (occluder) OR (device)) AND ((embolization) OR (dislodge∗) OR (migrat∗))] identified 14 individual case reports and one case series describing PFO device embolization events.

Timing appears critical: PFO occluder embolization discovered shortly after implantation can often be safely managed percutaneously,^1^^,^^3^, ^4^, ^5^, ^6^ whereas delayed retrieval may carry a higher risk of aortic injury. This is illustrated both by our case and by a report by Berk TA et al,^7^ where surgical extraction was similarly complicated by aortic dissection. This is likely due to the development of adhesions between the migrated device and the aortic wall, as demonstrated by our histological study. Additional contributing factors may include subclinical aortopathy, suggested by the mildly dilated ascending aorta and a family history of dissection.

Several reports have described failed attempts to retrieve embolized devices through short sheaths ending in the femoral artery, necessitating surgical cutdown.^2^^,^^4^ Davies et al^2^ described a case where an embolized Amplazer Cribrifrom Septal Occluder could not be retrieved past the iliac bifurcation using a short 20-F sheath and ultimately required surgical removal, complicated by distal embolization. In contrast, our use of a long 14-F sheath facilitated capture within a large-caliber vascular segment, allowing complete percutaneous retrieval.

Multiple reports^1^^,^^3^^,^^5^^,^^6^^,^^8^ have highlighted the advantages of using long sheaths, even more so than larger diameters, in reducing both procedural difficulty and risk of vascular trauma. Our 14-F sheath was 5-F larger than the original 9-F sheath, consistent with prior recommendations to use retrieval sheaths 2-F larger.^9^

In rare cases, conservative management may be a valid alternative. One patient with device embolization detected 12 months post implant remained asymptomatic at 24 months under dual antiplatelet therapy.^8^

Given the lack of randomized data and the rarity of this complication, the decision to pursue percutaneous, surgical, or conservative treatment should be individualized and guided by a multidisciplinary heart team discussion, with detailed patient counseling being essential.

## Conclusions

Endovascular retrieval of a PFO occluder embolized to the abdominal aorta is feasible and can be accomplished using a long sheath 2- to 5-F sizes larger than the original delivery sheath. However, delayed retrieval increases the risk of aortic injury due to device adherence to the vessel wall. Careful sizing, recognition of anatomical risk factors, and early postprocedural imaging may help prevent and detect this complication early on, avoiding the risks associated with delayed recovery.

## Funding Support and Author Disclosures

Dr Garot received proctor/consultant fees from Abbott, Boston Scientific, and Edwards Lifesciences. Mr Huel is an employee of Abbott Cardiovascular, France. All other authors have reported that they have no relationships relevant to the contents of this paper to disclose.

**Take-Home Messages**

• Aneurysmal interatrial septum and long patent foramen ovale tunnels are risk factors for patent foramen ovale occluder device embolization.
• Early follow-up imaging may help identify silent device migration before the development of adhesions between the embolized device and the vessel walls.
• When endovascular retrieval is necessary, a long sheath 2- to 5-F larger than the original delivery system is recommended to minimize procedural risk.

## Appendix

Video 1 Transesophageal Echocardiography Showing a Patent Foramen Ovale.

Video 2 Angiography Demonstrating Mobilization of the Embolized Patent Foramen Ovale Occlude (Arrow) From the Abdominal Aorta by Snaring the Screw of the Device Using a Tulip Snare Introduced into a 6F JR Catheter.

Video 3 Contrast Injection into the Abdominal Aorta Postretrieval Showing no Evidence of Aortic Dissection.

Video 4 Angiography Depicting the Occluder (Arrow) Being Retracted into the Sheath.

Video 5 Successful Retrieval of the Occluder (Arrow) Through the Sheath.

Video 6 Transesophageal Echocardiography After Repeat Patent Foramen Ovale Closure Demonstrating a Negative Bubble Test.
